# Validation of a classification and scoring system for the diagnosis of laryngeal and pharyngeal squamous cell carcinomas by confocal laser endomicroscopy

**DOI:** 10.1016/j.bjorl.2021.06.002

**Published:** 2021-07-20

**Authors:** Matti Sievert, Konstantinos Mantsopoulos, Sarina K. Mueller, Robin Rupp, Markus Eckstein, Florian Stelzle, Nicolai Oetter, Andreas Maier, Marc Aubreville, Heinrich Iro, Miguel Goncalves

**Affiliations:** a Friedrich-Alexander-Universität Erlangen-Nürnberg, University Hospital, Department of Otorhinolaryngology, Head and Neck Surgery, Erlangen, Germany; b Institute of Pathology, Friedrich-Alexander-Universität Erlangen–Nürnberg, University Hospital, Erlangen, Germany; c Friedrich-Alexander-Universität Erlangen–Nürnberg, University Hospital, Department of Maxillofacial Surgery, Erlangen, Germany; d Pattern Recognition Lab, Computer Science, Friedrich-Alexander-Universität Erlangen–Nürnberg, Erlangen, Germany; e Institute of Image Understanding and Medical Application of Artificial Intelligence, Technische Hochschule, Ingolstadt, Germany; f University Hospital Aachen, RWTH Aachen University, Department of Otorhinolaryngology, Head and Neck Surgery, Germany

**Keywords:** Confocal laser endomicroscopy, Squamous cell carcinoma, Head and neck cancer, Diagnosis

## Abstract

**Introduction:**

Confocal laser endomicroscopy is an optical imaging technique that allows in vivo, real-time, microscope-like images of the upper aerodigestive tract’s mucosa. The assessment of morphological tissue characteristics for the correct differentiation between healthy and malignant suspected mucosa requires strict evaluation criteria.

**Objective:**

This study aims to validate an eight-point score for the correct assessment of malignancy.

**Methods:**

We performed confocal laser endomicroscopy between March and October 2020 in 13 patients. 197 sequences (11.820 images) originated from the marginal area of pharyngeal and laryngeal carcinomas. Specimens were taken at corresponding locations and analyzed in H&E staining as a standard of reference. A total of six examiners evaluated the sequences based on a scoring system; they were blinded to the histopathological examination. The primary endpoints are sensitivity, specificity, and accuracy. Secondary endpoints are interrater reliability and receiver operator characteristics.

**Results:**

Healthy mucosa showed epithelium with uniform size and shape with distinct cytoplasmic membranes and regular vessel architecture. Confocal laser endomicroscopy of malignant cells demonstrated a disorganized arrangement of variable cellular morphology. We calculated an accuracy, sensitivity, specificity, positive predictive value, and negative predictive value of 83.2%, 81.3%, 85.5%, 86.7%, and 79.7%, respectively, with a κ-value of 0.64, and an area under the curve of 0.86.

**Conclusion:**

The results confirm that this scoring system is applicable in the laryngeal and pharyngeal mucosa to classify benign and malignant tissue. A scoring system based on defined and reproducible characteristics can help translate this experimental method to broad clinical practice in head and neck diagnosis.

## Highlights

• Confocal laser endomicroscopy enables real-time, non-invasive identification of malignancy in pharynx and larynx.
• Confocal laser endomicroscopy classification scores for the oral mucosa are also valid in pharynx and larynx.
• Sensitivity and specificity for carcinoma was 81.3% and 85.5%, respectively.
• Confocal laser endomicroscopy can be performed with substantial interrater agreement (k = 0.64).
• Presently confocal laser endomicroscopy can aid intraoperative assessment but does not replace histology.

## Introduction

Histological examination is needed to diagnose pharyngeal and laryngeal carcinoma and intraoperative aid assessment of free margins during oncologic surgery. Various optical methods, such as confocal laser endomicroscopy (CLE), optical coherence tomography, and near-infrared fluorescence endoscopy, have been shown to perform an optical biopsy, i.e., differentiate benign from malign lesions.^1^, ^2^, ^3^, ^4^, ^5^ CLE provides a magnifying power up to 1000 times by using fluorescein to outline intercellular spaces and depict tissue architecture and has been extensively applied in the evaluation of mucosa lesions in gastroenterology, urology, and pneumology.^6^, ^7^, ^8^, ^9^, ^10^, ^11^ CLE image interpretation has a moderate learning curve, and regarding the interrater reliability, there is inconsistency in the literature.^11^, ^12^, ^13^, ^14^ In order to gain acceptance in the clinical setting, however, result consistency is needed. A classification system was developed and validated in the head and neck region to the oral mucosa, but there is no data regarding this scoring sheet in the pharyngeal or laryngeal region.^15^ This scoring system prioritizes homogeneity and intercellular gap changes in the tissue architecture and the inconsistency of cell morphology. Fluorescein leakage and vessel shape are also taken into consideration.^15^ Our main objective was to evaluate its diagnostic metrics in identifying pharyngeal and laryngeal carcinoma compared to the gold standard of histopathological examination.

## Materials and methods

### Study design

We conducted this prospective pilot study at a tertiary hospital and academic cancer center. The study was approved by the local institutional ethics committee (Approval protocol number 60_14 B) and carried out following the Declaration of Helsinki. We obtained written, informed consent from all study participants.

### Eligibility criteria

Patients with confirmed head and neck squamous cell carcinoma (HNSCC) and planned open pharyngectomy and/or laryngectomy for tumor resection between September and October 2020. Exclusion criteria were a prior head and neck cancer, prior radiation in the head and neck area, distant metastasis, pregnancy, thyroid dysfunction, severe kidney failure, allergy to fluorescein, and patients under the age of 18.

### Technical details

We performed intraoperative image acquisition using a GastroFlex probe combined with a 488 nm Cellvizio laser scanning system (Mauna Technologies, Paris, France). The 2.6 mm diameter probe has a field of view of 240 μm and a resolution of 1 μm. We used 5 mL, fluorescein Alcon, 10% (Alcon Pharma, Freiburg, Germany) as an optical imaging dye for staining intercellular spaces and cytoplasmic components. This enabled outline visualization and structural analysis of cellular tissue and the loss of nuclear polarity and abnormal microvasculature patterns, which are usually present in tumor tissue.^16^, ^17^ This method enables the emission of laser light with a penetration depth of 55–65 μm. The reflected fluorescence light is refocused for detection. A pinhole excludes scattered or reflected light from other depth planes, thus enabling an increased spatial resolution.^18^

### Surgical procedure and intraoperative imaging

All patients underwent radical tumor resection. This provided an optimal exposure of the tumor margin for the acquisition of CLE sequences. Tumor resection was followed in every case by biopsy of the surrounding tissue to confirm safe margins and R0 resection. This included biopsy of surrounding larynx and hypopharynx in cases of oropharyngeal carcinoma as, well as every other combination depending on the size of the tumor. For this reason and considering the continuum of mucosa in these three areas we do not separate artificially the groups for the purpose of examination of cellular examination with CLE but perform this examination from the described surgical perspective. For this purpose, 2.5 mL fluorescein Alcon 10% was injected intravenously. After two minutes, the laser scanning unit was initiated. To enhance imaging quality, we applicated additional 2.5 mL after five minutes of examination.^19^ We correlated the CLE sequences with histopathology’s gold standard. For this purpose, we obtained a specimen from the investigated area after every single CLE sequence. Histologically confirmed healthy epithelium was obtained from the tumor bed, which is both essential for the standard confirmation of complete tumor resection as well as to validate our classification system. The histopathological assessment followed a standard protocol with hematoxylin and eosin (H&E) staining. After completing the CLE examination, we performed the tumor resection with a macroscopic safety margin of 1 cm. Our and international treatment standards were not altered or influenced in any way by the use of CLE.

### Scoring system (“Diagnosing Oral Cancer” – DOC-Score)^15^

**Figure 1:**
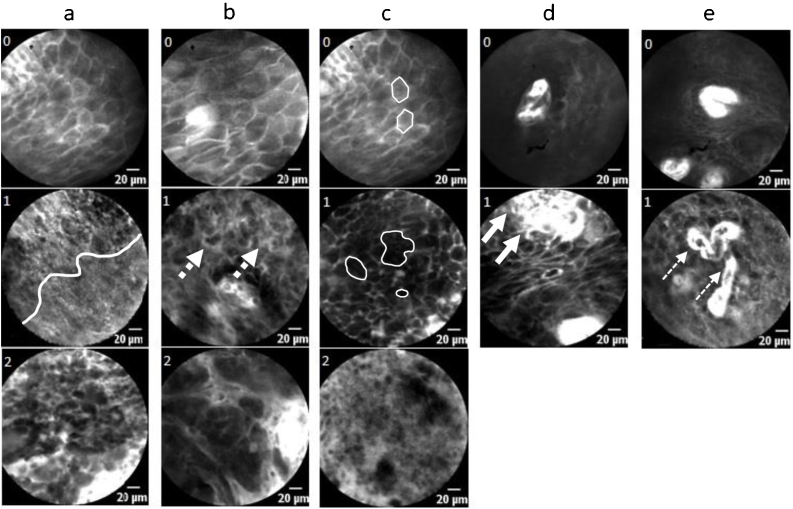
Graphical illustration of the DOC-Score. (a) Homogeneity: 0 – completely organized tissue, 1 – partially disorganized architecture (white line separates two areas), 2 – completely disorganized tissue; (b) Intercellular gaps: 0 – normal, 1 – mutaded (dotted arrows), 2 – non-existent; (c) Cell morphology (outlined by lines): 0 – honeycomb shape, 1 – different sizes/shapes/grey levels, 2 – dark and small cells/blurry and cloudy image/black spot (cell cluster); (d) Fluorescence leakage: 0 – regular, 1 – amplified (arrows: bright background); (e) Vessels: 0 – regular, 1 – irregular in shape/caliber/quantity (lined arrows: aberrant vessel course); x ≥ 5 points = squamous cell carcinoma.

This scoring system, developed initially for oral cavity carcinoma, evaluates five different CLE images or video sequences (Fig. 1a–e): homogeneity of tissue architecture (a), intercellular gaps (b), cell morphology (c), fluorescence leakage (d), and vessel regularity (e). A maximum of 8 points is achievable, and a score of 5 or more defines carcinoma. Figure 1 demonstrates the scoring system. Changes regarding homogeneity, intercellular gaps, and cell morphology are prioritized (up to 2 points each), changes in fluorescence leakage and vessel architecture are taken into account with 1 point.

### Data analysis

The investigator edited CLE data postoperatively using Cellvizio Viewer software 1.6.2. A total of 41.118 CLE frames were viewed and evaluated according to their quality. For evaluating the DOC-Score, 11.820 frames in a total of 197 sequences (each of 5 s) of high quality and free of artifacts were selected. Each of these 197 sequences was classified as malignant or benign based on an intraoperatively obtained H&E sample. The 197 video sequences were presented to six medical professionals for assessment, blinded to the histological results, as well as to the intraoperative macroscopical appearance. The professionals consisted of five head and neck surgeons (S1–S5; three experienced and two inexperienced) and one pathologist with previous experience in this technique. Surgeons with experience in the technique were defined as having performed at least 20 CLE cases. The three experienced surgeons and the pathologist’s results were summarized to an expert group (S1–S3, P). In contrast, we subsumed the results of S4 and S5 to a non-expert group.

### Statistical analysis

We performed statistical analysis using SPSS version 22.0 (IBM SPSS Statistics for Windows, Version 22.0. Armonk, NY, USA). The sensitivity, specificity, positive predictive value (PPV), negative predictive value (NPV), and accuracy were calculated for each examiner. The inter-rater reliability/ agreement was tested using Cohen’s kappa and Fleiss kappa coefficient. We interpreted κ-values according to Landis and Koch.^20^ Values of κ between 0.0 and 0.20 are defined as low, between 0.21 and 0.40 as fair, between 0.41 and 0.60 as moderate, between 0.61 and 0.80 as substantial, and between 0.81 and 1.0 almost perfect. We performed a receiver operating characteristic (ROC) analysis to calculate the area under the curve (AUC) as a quality measure for the DOC-Score as a classifier. Its resulting sensitivity and specificity were compared with a chi-square. The independent Student t-test performed the comparison of metric variables. A p-value of less than p ≤ 0.05 was considered statistically significant.

## Results

### Patient characteristics

Between March and October 2020, we enrolled thirteen patients (one female and twelve male; mean age 61.9 years (SD = 5.8) to undergo laryngeal and/or pharyngeal in vivo CLE during planned transoral/transcervical tumor resection. In five cases, tumor resections involved the oropharynx, and in eight cases, tumor resections involved the hypopharynx and larynx. Patient characteristics, including stage, are shown in Table 1. In all cases, safe margin resection could be performed independent of the use of CLE.

**Table 1:** Tumor characteristics.

Case nº	Age (years)	Tumor Stage	Location	Grade	CLE frames (n)	Recording time (s)	Selected sequences (n)
1	57	pT2	Oropharynx	G2	2217	277	11
2	61	pT2	Oropharynx	G2	1960	245	15
3	51	pT3	Oropharynx	G3	2418	302	11
4	61	pT2	Oropharynx	G3	3498	437	17
5	61	pT3	Oropharynx	G3	2716	339	13
6	67	pT1	Larynx	G2	7586	948	10
7	60	pT1	Larynx	G2	2841	355	14
8	68	pT4a	Larynx	G3	1919	239	19
9	73	pT4a	Larynx	G3	2413	301	12
10	57	pT3	Larynx	G3	6513	814	31
11	69	pT3	Larynx/Hypopharynx	G3	2398	299	11
12	63	pT3	Larynx/Hypopharynx	G3	2896	362	14
13	57	pT3	Larynx/Hypopharynx	G3	1743	217	19

### CLE image acquisition

The average image acquisition time for each case was 6.6 min (SD =3.6 min). We acquired a total of 41.118 CLE frames, with a mean of 3.163 frames (SD = 1.729) in each surgical procedure. On average, 15.1 sequences (SD = 5.4) from each patient were selected for DOC analysis. Analysis of the corresponding histologic specimens from all 197 CLE sequences revealed normal epithelium in 91 sequences (46.2%) and HNSCC in 106 sequences (53.8%).

### Diagnostic metrics

The performance of the classification based on the DOC-Score is illustrated in Table 2. According to Oetter et al.,^15^ we considered a score of ≥5 points to be malignant. The examiners achieved a mean accuracy, sensitivity, specificity, PPV, and NPV ranging from 76% to 89%, 70% to 91%, 80% to 91%, 80% to 92%, and 70% to 90%, respectively. In summary, we could show a mean accuracy, sensitivity, specificity, PPV and NPV of 83.2% (95% CI: 81.0–85.3), 81.3% (95% CI: 78.0–84.2), 85.5% (95% CI: 82.3–88.4), 86.7% (95% CI: 84.2–88.9), and 79.7% (95% CI: 76.9–82.2). The observers’ overall agreement was calculated with a Fleiss` κ of 0.64 for the 197 videos and can therefore be considered substantial. In computing the inter-rater reliability in the selective analysis between two examiners, Cohens’ κ ranged from 0.46 to 0.80, with a percentage agreement level ranging from 73.1% to 89.8% (Table 3).

**Table 2:** Data analysis by the several reviewers.

Diagnostic metrics	P	S1	S2	S3	S4	S5	All
	Value (95% CI)	Value (95% CI)	Value (95% CI)	Value (95% CI)	Value (95% CI)	Value (95% CI)	Value (95% CI)
Accuracy	85.8% (80.1–90.3)	88.8% (83.6–92.9)	84.8% (79.0–89.5)	86.3% (80.7–90.8)	77.7% (71.2–83.3)	76.1% (69.6–81.9)	83.2% (81.0–85.3)
Sensitivity	83.0% (74.5–89.6)	86.8% (78.8–92.6)	89.0% (80.7–94.6)	91.5% (84.5–96.0)	75.5% (66.2–83.3)	69.8% (60.1–78.)	81.3% (78.0–84.2)
Specificity	89.0% (80.7–94.6)	91.2% (83.4–96.1)	81.1% (72.4–88.1)	80.2% (70.5–87.8)	80.2% (70.5–87.8)	83.5% (74.3–90.5)	85.5% (82.3–88.4)
PPV	89.8% (83.0–94.0)	92.0% (85.5–95.7)	80.2% (73.0–85.8)	84.3% (78.0– 89.1)	81.6% (74.3–87.2)	83.1% (75.3–88.8)	86.7% (84.2–88.9)
NPV	81.8% (74.6–87.3)	85.6% (78.4–90.6)	89.6% (82.6–94.0)	89.0% (81.1– 93.9)	73.7% (66.4–79.9)	70.4% (63.7–76.3)	79.7% (76.9–82.2)
ROC (AUC)	0.90 (0.86–0.94)	0.91 (0.87–0.95)	0.88 (0.84–0.93)	0.85 (0.81–0.90)	0.81 (0.76–0.86)	0.83 (0.78–0.88)	0.86 (0.84–0.88)

P, pathologist; S, surgeon; PPV, positive predictive value; NPV, negative predictive value; 95% CI, 95% confidence interval; ROC, receiver operating characteristic; AUC, area under the curve.

**Table 3:** Inter-observer agreement.

Observer pair	Cohens Kappa/Fleiss-Kappa^α^	Agreement (%)
S1 & S2	0.74	86.8
S1 & S3	0.66	83.3
S1 & S4	0.59	79.7
S1 & S5	0.60	80.2
S1 & P	0.80	89.8
S2 & S3	0.60	79.2
S2 & S4	0.65	82.7
S2 & S5	0.62	81.2
S2 & P	0.72	85.8
S3 & S4	0.46	73.1
S3 & S5	0.52	75.6
S3 & P	0.67	83.3
S4 & S5	0.73	86.3
S4 & P	0.59	79.7
S5 & P	0.62	81.2
S1–3 & P	0.69^α^	
S1–5 & P	0.64^α^	

Inter-observer agreement for observer pairs (Cohens Kappa) and for multiple Raters (Fleiss Kappa^α^) as well as percentual agreement.

S, surgeon; P, pathologist.

The average DOC-Score value of experts was 6.2 (SD = 2.3) points for histologically confirmed HNSCC and 2.0 (SD = 2.2) points for normal epithelium. In non-experts, the score was 5.3 (SD = 2.3) and 2.0 (SD = 2.4) points, respectively (*p* =  0.000). The selective analysis of the expert group reveals a mean sensitivity, specificity, PPV, and NPV of 83.0% (95% CI: 74.5–89.6), 89.0% (95% CI: 80.7–94.6), 89.8% (95% CI: 83.0–94.0), and 81.8% (95% CI: 74.6–87.3), respectively with an inter-rater reliability of κ = 0.69. In the non-expert group, a mean sensitivity, specificity, PPV, and NPV of 72.6% (95% CI: 66.1–78.5), 81.9% (95% CI: 75.5–87.2), 82.3% (95% CI: 77.2–86.5), and 72.0% (95% CI: 67.1–76.4) can be observed (κ = 0.73). The ROC analysis demonstrates an AUC of 0.88 for the expert group, compared to an AUC of 0.82 for the non-expert group. With a mean accuracy of 85.8% (95% CI: 80.1–90.3) and 76.9% (95% CI: 72.4–80.9) for experts and non-experts, we can confirm a significant difference between both groups (*p* < 0.001).

## Discussion

We report on the transferability of a CLE classification system^15^ developed for oral mucosa to the pharyngeal and laryngeal epithelium. Based on 197 sequences (11.820 images) blinded to the gold standard of histopathological examination, differentiation of benign and malign lesions was possible with an accuracy, sensitivity, and specificity, of 83.2%, 81.3%, 85.5%, respectively. The examination added around 15 min to standard operation time without any side effects after intravenous fluorescein application. Tissue architecture (2 points), intercellular gaps (2 points), cell morphology (2 points), fluorescence leakage (1 point), and vessel shape (1 point) were classified according to the DOC-Score. In this regard, we confirmed the 2.6 mm CLE probe provided sufficient spatial resolution and tissue contrast to distinguish cellular architecture, borders, and size to characterize healthy and malignant tissue in the intraoperative setting. Malignant tissue samples were classified on average with 6.2 points and benign epithelium with 2.0 points among all examiners. Interpretation of CLE images are subjective and a crucial factor in making a correct assessment that requires, up to now, the experience and expertise of specialists. In this study, we defined experts as having performed more than 20 examinations with CLE. Interrater variability has been described with varying results (fair to almost perfect), especially when non-experts performed the examination.^11^, ^12^, ^13^, ^14^

Oetter et al. developed and validated the DOC-Score based on 95 sequences (6224 images) to diagnose oral HNSCC through CLE.^15^ Using the DOC-Score, sensitivity and specificity of 95.3% and 88.9% for experts and 97.3% and 88.1% for non-experts was achieved. Inter-rater reliability (Fleiss` kappa) was 0.73 for experts and 0.814 for non-experts. Our values were lower (Sensitivity and Specificity of 83.2%, 82.1%, respectively) with an AUC of 0.88 for experts and 0.82 for non-experts. Interrater reliability was 0.73 for non-experts in our group, which was above the 0.64 calculated for the whole group and thus demonstrates the value of a systematic classification in a CLE-naive examiner. An accurate histopathological diagnosis of HNSCC is vital in guiding clinical management, and gold-standard for intraoperative assessment of free margins are adequate circumferential frozen sections. CLE images have a similar resolution to traditional H&E and could serve as valuable adjunct technology by potentially minimizing the need for intraoperative frozen section analysis. Pathological assessment of frozen sections and biopsy specimens does not typically involve the surgeon; however, a trained head and neck surgeon can perform CLE image interpretation for a real-time distinction of normal from abnormal tissue. The ability to sample the entirety of a tumor and surrounding tissue at the time of surgery may help guide the procedure's extent of resection. A pathologist (P), blinded to the histopathological examinations, also evaluated the CLE images. Interestingly the pathologist achieved an accuracy of 85.8%, very much comparable to the expert group (S1, S2, S3: 84.8%–88.8%) and better than the non-expert (S4, S5: 76.1%–77.7%), which underlines how principles used to classify histopathological slides are in some form adaptable to CLE images. Due to the physical properties of the technique, the penetration depth is fixed by 65 µm. Therefore, it was impossible to distinguish carcinoma in situ from invasive carcinoma since stromal invasion cannot be used as a criterion to differentiate between these two.^21^

A further possibility to objectivize CLE findings is utilizing automatic classification.^22^, ^23^, ^24^ Aubreville et al. showed that an approach based on transfer learning from intermediate endpoints within a pre-trained inception v3 network with preprocessing could reach an overall 94.8% accuracy, significantly improving overall performance over the traditional state of the art feature-based machine learning approaches.^22^ Automatic classification methods for CLE in the head and neck were developed for vocal cords and oral mucosa, but until this point, there is no available data for the pharyngeal and remaining laryngeal region. A prerequisite in developing such an automatic classification method based on deep learning-based approaches is acquiring large amounts of data and the correct labeling of such images in the healthy epithelium of a specific anatomic region and cancerous cells. Interestingly, compared to a previous study on the transferability of automatic classification based on algorithms trained with images of vocal cords applied on the oral cavity and vice versa, an accuracy of 68.5% and 89.5% was found.^24^ This suggests that the epithelium of these areas and the carcinomas that arise are similar on CLE; however, there seem to be significant differences that limit its detection rate. The same difficulties can also be pointed to the blinded examiners, mostly in the oral cavity and on vocal cords/larynx. The present work increases the information and knowledge of, until now, less examined anatomic regions and confirms the clinical criteria used to classify the oral cavity lesions are also mostly valid in the pharyngeal and laryngeal regions. Some differences are assumed since the diagnostic metrics are admittedly good but still not as accurate as for the oral cavity.^15^ Starting from the DOC-Score basis, some adaption of these criteria is probably required if it is to be applied to the whole head and neck region and should be assessed in further studies.

The absence of classification criteria and small data available limit CLE’s clinical application in head and neck until this day. However, we demonstrated that the DOC-Score provides a very good basis for establishing the head and neck region's diagnosis, despite the better performance in the oral cavity, which was the region it was originally developed for. CLE has, however, as a method with experimental status, limitations intrinsic to this technology and independent of the classification system used. As the probe-based CLE has only a penetration depth of 55–65 μm, it is not possible to assess the deeper margins in muscle and fascia. Indeed, mucosal epithelium is at its thinnest (floor of the mouth) over 100 μm, which formally makes the differentiation between carcinoma in situ and invasive carcinoma, as well as infiltration of submucosa, muscle and fascia very difficult.^11^, ^25^ Direct evaluation of these deeper layers with CLE will need to be investigated in the future. CLE has a considerable potential to aid intraoperative characterization of mucosal regions in the head and neck, dependent on further validation in clinical trials.

## Conclusion

By differentiating in real-time, in vivo healthy mucosal cells from squamous cell carcinoma with a sensitivity and specificity of 81% and 86%, respectively, CLE is a promising imaging technology that may improve the non-invasive characterization of HNSCC. A standardized classification system could improve diagnostic accuracy and consistency of results among examiners.

## Funding

This project was supported by the German Research Foundation (DFG, Deutsche Forschungsgemeinschaft) with grant number GO 3182/2-1, MA 4898/17-1, OE 743/1-1, STE 1877/7-1; Project Number 439264659.

## Conflicts of interest

The authors declare no conflicts of interest.
